# The Effects of Health Education on the Awareness of Antimicrobial Resistance Among High School Students in Riyadh, Saudi Arabia During 2023: A Quasi-experimental Study

**DOI:** 10.7759/cureus.41639

**Published:** 2023-07-10

**Authors:** Khalid S Almutairi, Ezzuddin A Okmi, Sabah S Alnofaiei, Waleed K Alshamari, Sultan H Almutairi, Sulaiman I Alsuwailem, Eid H Alkhaldi

**Affiliations:** 1 Preventive Medicine, Ministry of Health, Riyadh, SAU; 2 Respiratory Infectious Diseases Prevention and Control, Saudi Public Health Authority, Riyadh, SAU; 3 Psychology, Prince Sultan Military Medical City, Riyadh, SAU; 4 Preventive Medicine, King Fahad Military Medical City, Damman, SAU; 5 Preventive Medicine, Saudi Public Health Authority, Riyadh, SAU

**Keywords:** high school students, educational intervention, awareness, antibiotic resistance, antimicrobial resistance

## Abstract

Introduction

Antimicrobial resistance (AMR) is a major health threat, recently declared a crisis by the WHO, and recognized as one of the top 10 threats to global health. One of the strategies to curb AMR is interventional education to raise awareness. Therefore, this study evaluated the impact of interventional education on awareness of antimicrobial resistance among high school students in Riyadh, Saudi Arabia.

Methods

This was a quasi-experimental study that included 120 high school students as a control group and 120 students as the experimental group. It used a questionnaire pre- and post-educational intervention, which was a lecture by trained healthcare workers. Pearson’s Chi-square test and ANOVA were used to assess the effects of the intervention, and the p-value was set at <0.05 for significance.

Results

Over half (53.4%) of the controls reported no prior exposure to an antibiotic awareness campaign, compared to 46.6% in the intervention (experimental) group. Half of the participants in both groups were without a family relative who works in the healthcare sector. Almost half (51.2%) in the intervention group used antibiotics compared to 48.2% of controls; 53.3% in the control group reported self-medication compared to 46.7% in the intervention group. We found a statistically significant increase in the overall mean scores regarding knowledge of antibiotics resistance before and after the education intervention in the control group (p<0.001) and intervention group (p<0.001). Post-intervention, there was a significant reduction in the mean scores of misbeliefs about antibiotic use (p<0.001). We found an improvement in the perception scores toward AMR and antibiotic resistance post-educational intervention (P=0.008), and the perception difference remained significant between the two study groups (p=0.002).

Conclusion

These findings showed that interventional education effectively raises awareness, knowledge, and perceptions toward AMR. Therefore, public health, medical, and scientific professionals in Saudi Arabia are urged to emphasize education to fight AMR, in addition to other strategies.

## Introduction

Antimicrobial resistance (AMR) occurs when the medications active against infections, such as antibiotics, antiviral, antifungals, and antiparasitic, including antimalarials, lose the ability to prevent or treat the underlying microorganism [1]. AMR was recently declared a disaster, a crisis for the health system, and one of the top 10 global health threats encountering humanity, according to the World Health Organization (WHO) [2]. In 2019, AMR was associated with approximately 5 million deaths, including 1.27 million deaths from bacterial AMR [3]. A high prevalence of AMR impacts the quality of care as it depends on more expensive second and third-line medications and hospitalization, raising costs and limiting the accessibility of treatment in resource-limited areas, especially in low and middle-income countries [4]. In the worst-case scenarios, diseases become resistant to all types of medications, increasing morbidity and mortality [5]. It is estimated that if the AMR continues to rise, it could lead to 10 million death per year and a reduction in the Gross Domestic Product (GDP) by 2-3.5%. It could also cost 100 trillion US dollars and increase the incidence of extreme poverty by 2050 [6].

Strategies such as public health awareness, vaccination, sanitation, hygiene, and safe water provision, vital in infection prevention and transmission control, also play a critical role in preventing antimicrobial resistance [7-10]. This highlights the contribution of public health awareness of these strategies in decreasing AMR at all levels. However, globally, there are misunderstandings and poor public knowledge of AMR and its development, and misconceptions about AMR [11]. Without public health awareness, efforts to combat AMR will not be effective. On the other hand, raising awareness could be cost-effective and lead to the reduction of AMR. In Belgium, campaigns to reduce the misuse of antibiotics during the winter season were found to decrease outpatient antibiotic use by 12.8% for twice-a-day use and 42.8% for thrice-a-day use, and decreased antibiotic costs and reduced AMR [12]. A study evaluating the pre-service education on AMR and antimicrobial stewardship (AMS) among healthcare workers showed low coverage of the topic and poor depth in most curricula [13]. A study conducted in Myanmar found that only 56.3% of participants from the general public have heard of AMR, only 0.9% correctly answered all five questions about antibiotics, and 58.5% purchased antibiotics without a prescription [14].

Studies conducted in Saudi Arabia have found that there is a lack of awareness about AMR and the proper usage of antibiotics among the general population, with only 40.5% having sufficient knowledge about AMR, while 75% of medical and health-related science students and 71.5% of community pharmacists followed good practices in dispensing antibiotics [15-17]. Research has indicated that education is an effective and cost-saving strategy to improve the correct antibiotic use and reduce AMR [18]. However, there is no study done in Saudi Arabia to evaluate the effectiveness of education on AMR. Therefore, this study aimed to evaluate the effect of educational intervention on awareness of antimicrobial resistance in Saudi Arabia.

## Materials and methods

Study design and sampling

A quasi-experimental study was conducted on high school students aged between 16 and 18 years in Riyadh, Saudi Arabia. Students with mental illness or who were in a special education program were excluded. The selected participants were divided into two groups (experimental and control groups), with 120 students for each group. Based on 95% confidence, power of 80%, and a dropout rate of 10%, the sample size estimation was 240**.** We selected three schools, and students from each school were chosen from level 1A, level 2B, and level 3A. These are three high school education levels in Saudi Arabia, and each level has classrooms: Classroom A, Classroom B, and Classroom C. 

Data collection tool

We used a validated questionnaire from a previously published article [19] with permission from the principal investigator. The primary questionnaire, developed in English, was translated into Arabic by a professional translator and reviewed by two experts in medical research. The questionnaire consists of five parts: Part I is about demographic characteristics; Part II inquires about background data on antibiotic use; Part III is about knowledge towards antibiotic resistance; Part IV assesses knowledge of antibiotic use; and Part V assesses perception toward antibiotic use. The questionnaire had a Cronbach’s alpha value of more than 0.7 in the pilot test, and further details are available in a study by Thong et al. [19].

Interventions

The pre-intervention assessment using a questionnaire was conducted to identify baseline knowledge for both groups. The educational intervention was delivered to the experimental group in the form of a lecture by a trained healthcare worker using materials sourced from the United States Centers for Disease and Prevention and Control (CDC) and the WHO about AMR and antibiotic use [20,21]. A placebo education, also in the form of a lecture by a trained healthcare worker, was given to the control group using materials sourced from the Saudi Ministry of Health about the benefits of physical activity. Each educational session lasted approximately 15 minutes for both groups. Fifteen minutes of education regarding antimicrobial resistance using CDC and WHO materials were found to be effective [19]. Immediate post-intervention assessment was done for both groups using the same questionnaire to evaluate the impact of the interventions.

We could not conduct a two-week post-intervention assessment due to legal restrictions in Saudi Arabia that prevented us from keeping students' contact details and names for future identification of the same participants.

Ethical approval

Written consent was requested from the participants and their parents. No personal identifying information was collected since the questionnaire was anonymous. Ethical approval from the institutional review board of King Fahad Medical City was received (Ref. No: 23-205E) and permissions from schools were obtained before conducting this study.

Data analysis

Data analysis was performed using IBM SPSS Statistics, Version 27 (IBM Corp, Armonk, NY, USA). Continuous variables were reported during descriptive tests using mean ± SD (Standard deviation), and categorical variables were expressed in frequency (percentage). For analytical tests, Pearson’s Chi-square test and ANOVA were performed to evaluate the effects of the intervention for both the intervention and control groups. The Kolmogorov-Smirnov test was performed to check the normality of the variables. The p-value was set at <0.05 for statistical significance.

## Results

The characteristics of the participants in the study are shown in Table 1. In the control group, there were 66 males (55%), while in the intervention group, there were 62 males (51.7%). In the control group, 53.4% of participants reported no prior exposure to an antibiotic awareness campaign, while 44.6% reported prior exposure. In the intervention group, 46.6% of participants reported no prior exposure, while 55.4% reported prior exposure.

**Table 1:** Characteristics of the participants Values represent the number (percentage). The p-value was calculated using Pearson’s Chi-square test.

Variables		Control group	Intervention group	Total	p-value
Gender	Male	66 (55.0%)	62 (51.7%)	128 (53.5%)	0.605
	Female	54 (45.0%)	58 (48.3%)	112 (46.7%)	
Have you been exposed to an antibiotic awareness campaign before?	No	79 (65.8%)	69 (57.5%)	148 (61.7%)	0.184
	Yes	41 (34.2%)	51 (42.5%)	92 (38.3%)	
Are you or any of your first-degree family members’ occupations healthcare related?	No	55 (45.8%)	55 (45.8%)	110 (45.8%)	0.999
	Yes	65 (54.2%)	65 (54.2%)	130 (54.2%)	

In both control and intervention groups, half of the participants did not have a healthcare-related occupation, and none of their family relatives had one, whereas the other half had a healthcare-related occupation. There were no significant differences between the control and intervention groups regarding gender distribution, exposure to antibiotic awareness campaigns, and healthcare-related occupations (all p>0.05).

Table 2 shows the participants' responses regarding their antibiotic uptake practices. Concerning the antibiotics taken during the lifetime, in the control group, 52.6% of the participants claimed to have never consumed antibiotics, while 47.4% made the same statement in the intervention group. In contrast, 48.5% of the control group reported antibiotic usage, whereas 51.2% of the intervention group admitted it.

**Table 2:** Antibiotics uptake practices by the participants Values represent the number (percentage). The p-value was calculated using Pearson’s Chi-square test.

Variables		Control group	Intervention group	Total	p-value
Have you taken antibiotics before in your lifetime?	No	40 (52.6%)	36 (47.4%)	76 (100.0%)	0.579
	Yes	80 (48.8%)	84 (51.2%)	164 (100.0%)	
When was your last antibiotic taken?	More than 1 year	11 (37.9%)	18 (62.1%)	29 (100.0%)	0.623
	Within 1 month	31 (50.0%)	31 (50.0%)	62 (100.0%)	
	Within 1 year	6 (46.2%)	7 (53.8%)	13 (100.0%)	
	Within 6 months	32 (52.5%)	29 (47.5%)	61 (100.0%)	
How frequently do you consume antibiotics in a year?	At least once a month	26 (61.9%)	16 (38.1%)	42 (100.0%)	0.160
	At least once a year	5 (31.3%)	11 (68.8%)	16 (100.0%)	
	At least once every 3 months	15 (42.9%)	20 (57.1%)	35 (100.0%)	
	At least once every 6 months	23 (52.3%)	21 (47.7%)	44 (100.0%)	
	Less than once a year	11 (39.3%)	17 (60.7%)	28 (100.0%)	
Have you self-medicated with the antibiotic for fever and sore throat without consulting a doctor?	No	31 (42.5%)	42 (57.5%)	73 (100.0%)	0.168
	Yes	49 (53.3%)	43 (46.7%)	92 (100.0%)	

Of those who used antibiotics, most (62.1%) in the intervention group had used antibiotics for the last time more than a year ago and most (50.0%) in the control group had used the antibiotics a month ago, which is similar percentages of the users in the intervention group last month. Most (61.9) participants in the control group used antibiotics at least once a month, followed by those who used them at least every 6 months (52.3%). In the intervention group, over two-thirds (68.8%) used antibiotics at least once a year.

In the control group, 42.5% of participants denied self-medication with antibiotics, whereas, in the intervention group, 57.5 % shared the same stance. By contrast, 53.3 % of the participants admitted to self-medication in the control group, whereas 46.7% in the intervention group made a similar confession.

There was no significant difference between the two groups in terms of the lifetime uptake of antibiotics for all participants (p=0.579). There were no significant differences between the two groups in terms of the last time for taking antibiotics among those who reported taking them, frequency of taking antibiotics in a year, and self-medication with antibiotics (all p>0.05).

The answers to questions assessing the participants' knowledge of antibiotic resistance causes are shown in Table 3. Before the education intervention, the control group had an antibiotic resistance definition knowledge mean score of 3.30 ± 0.874, while the intervention group had a significantly higher mean score of 3.51 ± 0.867 (p<0.001). After the education intervention, the control group's knowledge mean score slightly increased to 3.43 ± 0.953, while the intervention group's mean score significantly rose to 4.04 ± 1.032. There was a statistically significant difference in the mean score before and after the education intervention for both groups (p<0.001). Also, there was a statistically significant difference in the mean score between the control and intervention groups (p<0.001).

**Table 3:** Participants’ knowledge of causes of antibiotic resistance Values represent mean ± SD. P-value 1 represents the comparison within the measurements, whereas p-value 2 represents the comparison between the two study groups. Both were calculated by repeated measured ANOVA. *Significant. **Highly significant

Questions	Control group		Intervention group		p-value 1	p-value 2
	Pre-intervention	Post-intervention	Pre-intervention	Post-intervention		
Antibiotic resistance happens when the antibiotic loses its ability to cure a bacterial infection.	3.30 ± 0.874	3.43 ± 0.953	3.51 ± 0.867	4.04 ± 1.032	<0.001**	<0.001**
Taking leftover antibiotics from previous infections may cause antibiotic resistance.	3.25 ± 0.776	3.30 ± 1.021	3.25 ± 0.765	3.73 ± 1.296	0.006**	0.035*
Treatment may fail if you do not finish antibiotics as instructed by doctors.	3.97 ± 0.955	3.66 ± 1.029	3.80 ± 1.061	4.23 ± 0.939	0.408	0.054
Using antibiotics without consulting a doctor or purchasing directly from the pharmacy without a prescription may cause antibiotic resistance.	3.28 ± 1.144	3.19 ± 1.114	3.21 ± 1.071	3.84 ± 1.353	0.004**	0.025*
Overall mean	3.54 ± 0.562	3.40 ± 0.596	3.42 ± 0.619	3.96 ± 0.836	<0.001**	<0.001**

Concerning the knowledge that taking leftover antibiotics is a cause of antibiotic resistance, the control group's mean score before the intervention was 3.25 ± 0.776, which increased slightly to 3.30 ± 1.021 after the intervention. In contrast, the intervention group's mean score was 3.25 ± 0.765 before the intervention and significantly improved to 3.73 ± 1.296 after the intervention. There was a statistically significant difference in the mean score before and after the education intervention (p=0.006) and the mean score between the control and intervention groups (p=0.035).

Regarding the knowledge of the consequences of not completing a full course of antibiotics as instructed by doctors, the control group's mean score before the intervention was 3.97 ± 0.955, which decreased to 3.66 ± 1.029 after the intervention. Similarly, the intervention group's mean score before the intervention was 3.80 ± 1.061, which increased to 4.23 ± 0.939 after the intervention. However, no significant difference was found between the mean score before and after the education intervention nor between the two study groups (p>0.05).

The control group's knowledge mean score of the misuse of antibiotics without consulting a doctor as the cause of resistance before the intervention was 3.28 ± 1.144, which decreased to 3.19 ± 1.114 after the intervention. That of the intervention group before the intervention was 3.21 ± 1.071, which increased to 3.84 ± 1.353 after the intervention. There was a statistically significant difference in the mean score before and after the education intervention (p=0.004) and the mean score between the control and intervention groups (p=0.025).

Overall, the mean scores for all participants' knowledge of antibiotics resistance were higher in the intervention group than in the control group, both before and after the intervention (p<0.001). There was a statistically significant difference between the mean score before and after the education intervention and between the two study groups (p<0.001).

Participants' knowledge scores of antibiotic use are shown in Table 4. Regarding the belief that antibiotics could cure viral infections, before the intervention, the control group had a mean score of 4.03 ± 0.961, which decreased to 3.93 ± 0.954 after the intervention. In contrast, the intervention group had a lower mean score of 3.63 ± 1.182 before the intervention, significantly decreasing to 2.49 ± 1.517 after the intervention. There was a statistically significant difference in the mean score before and after the education intervention and between the two study groups (p<0.001).

**Table 4:** Participants’ knowledge of antibiotic use Values represent mean ± SD. P-value 1 represents the comparison within the measurements, whereas p-value 2 represents the comparison between the two study groups. Both were calculated by repeated measured ANOVA. *Significant. **Highly significant.

Questions	Control group		Intervention group		p-value 1	p-value 2
	Pre-intervention	Post-intervention	Pre-intervention	Post-intervention		
Viral infection can be cured by taking antibiotics.	4.03 ± 0.961	3.93 ± 0.954	3.63 ± 1.182	2.49 ± 1.517	<0.001**	<0.001**
Taking antibiotics early can prevent me from infection/illness	3.34 ± 1.278	3.28 ± 1.069	3.05 ± 1.122	3.07 ± 1.945	0.753	0.061
Taking antibiotics for common cough and wheezing can help me recover faster than not taking any antibiotics.	3.51 ± 1.149	3.68 ± 0.909	3.59 ± 1.056	2.84 ± 1.243	0.003*	<0.001**
Taking a low dose of antibiotics is better than not taking any dose.	3.57 ± 0.983	3.30 ± 1.082	3.58 ± 0.992	2.93 ± 1.196	<0.001**	0.101
Antibiotics-resistant bacteria can be spread from one person to another.	3.67 ± 1.106	3.48 ± 1.012	3.53 ± 0.978	4.05 ± 1.129	0.059	0.046*
Overall mean	3.63 ± 0.644	3.53 ± 0.594	3.47 ± 0.688	3.08 ± 0.713	<0.001**	<0.001**

The control group's mean score about believing that taking antibiotics early can prevent infection or illness before the intervention was 3.34 ± 1.278, decreasing to 3.28 ± 1.069 after the intervention. Similarly, the intervention group's mean score before the intervention was 3.05 ± 1.122, increasing to 3.07 ± 1.945 after the intervention. However, these slight differences between pre-and post-intervention did not show a significant difference within or between groups (p>0.05).

The study also examined the misconception that taking antibiotics for common cough and wheezing can help recover faster compared to not taking any antibiotics. The control group's mean score before the intervention was 3.51 ± 1.149, which increased to 3.68 ± 0.909 after the intervention. In contrast, the intervention group's mean score before the intervention was 3.59 ± 1.056, significantly decreasing to 2.84 ± 1.243 after the intervention (p=0.003). There was also a highly significant difference in the mean score between the control and intervention groups (p<0.001).

Though there was a statistically significant decrease in the mean scores regarding the belief that taking a low dose of antibiotics is better than not before and after the education intervention (p<0.001) for both groups, there was no statistically significant difference in the mean score between the control and intervention groups (p=0101).

When evaluating the belief that antibiotic-resistant bacteria can be spread from one person to another. The control group had a mean score of 3.67 ± 1.106 before the intervention, which decreased to 3.48 ± 1.012 after the intervention. In contrast, the intervention group had a mean score of 3.53 ± 0.978 before the intervention, which increased to 4.05 ± 1.129 after the intervention. A slightly significant difference was found between the two study groups (p=0.046), but no statistically significant difference in the mean score before and after the education intervention (p=0.059).

Overall, the mean scores for knowledge about antibiotics use were generally higher in the control group before the intervention compared to the intervention group (p<0.001), and significantly increased post-intervention in the same group (p<0.001).

Participants' perceptions scores towards antibiotic resistance are shown in Table 5. Regarding the belief that individuals who take antibiotics should stop their treatment earlier than recommended when symptoms improve, the control group had a mean score of 3.23 ± 1.203 before the intervention, which decreased slightly to 3.21 ± 1.187 after the intervention. In comparison, the intervention group initially had a higher mean score of 3.63 ± 1.182, significantly decreasing to 2.66 ± 1.470 (p= 0.001) after the intervention. However, there was no significant difference in the mean score between the control and intervention groups (p=0.074).

**Table 5:** Participants' perceptions towards antibiotic resistance Values represent mean ± SD. P-value 1 represents the comparison within the measurements, whereas p-value 2 represents the comparison between the two study groups. Both are calculated by repeated measured ANOVA. *Significant. **Highly significant.

Questions	Control group		Intervention group		p-value 1	p-value 2
	Pre-intervention	Post-intervention	Pre-intervention	Post-intervention		
Individuals taking antibiotics should stop their treatment earlier than recommended when symptoms improve.	3.23 ± 1.203	3.21 ± 1.187	3.63 ± 1.182	2.66 ± 1.470	0.001**	0.074
A doctor shall always prescribe antibiotics if someone is having fever, sore throat or runny nose.	3.61 ± 1.222	3.51 ± 1.069	3.58 ± 1.287	3.24 ± 1.408	0.028*	0.201
Antibiotics should be made available without doctor’s prescription.	2.43 ± 1.282	2.71 ± 1.268	2.71 ± 1.262	1.89 ± 1.539	0.453	<0.001**
I am not worried about the impact of antibiotic resistance on my health and my family.	2.35 ± 1.128	2.59 ± 1.178	2.39 ± 1.106	2.05 ± 1.339	0.604	0.049*
There is not much that I can do to stop antibiotic resistance.	2.92 ± 1.018	3.03 ± 1.045	2.91 ± 0.991	2.87 ± 1.049	0.590	0.389
Overall mean	2.91 ± 0.662	2.99 ± 0.662	2.89 ± 0.640	2.54 ± 0.794	0.008**	0.002**

Regarding the belief that antibiotics should be made available without a doctor's prescription, the control group had a mean score of 2.43 ± 1.282 before the intervention, which increased to 2.71 ± 1.268. In contrast, the intervention group had a mean score of 2.71 ± 1.262 before the intervention, significantly decreasing to 1.89 ± 1.539 after the intervention (p<0.001). However, no statistically significant difference in the mean score before and after the education intervention (p= 0.453).

Regarding the worry about the impact of antibiotic resistance on their health and their family, the control group had a mean score of 2.35 ± 1.128 before the intervention, which increased to 2.59 ± 1.178 after the intervention. Similarly, the intervention group had a mean score of 2.39 ± 1.106 before the intervention, which decreased to 2.05 ± 1.339 after the intervention. The two study groups showed a slightly significant difference (p=0.049), without any significant difference in the mean score before and after the education intervention (p=0.604).

The control group had an overall mean score of 2.91 ± 0.662 before the intervention, which increased to 2.99 ± 0.662 after the intervention. In comparison, the intervention group had a mean score of 2.89 ± 0.640 before the intervention, decreasing to 2.54 ± 0.794 after the intervention. There was a statistically significant difference between the mean score before and after the education intervention (p=0.008) and the difference remained significant between the two study groups (p=0.002).

## Discussion

Antimicrobial resistance (AMR) is a life-threatening public health burden worldwide due to resistant microorganisms due to antibiotic abuse and overuse [11,14,17]. It was included in the 10 top global public health threats facing humanity by the WHO [1,21]. Several approaches have been developed to address this problem, including educational programs designed to raise awareness, knowledge, and perception among medical personnel, patients, and the general public [18]. This is the first study conducted in Saudi Arabia to assess the effectiveness of educational interventions on AMR awareness.

We found that educational intervention significantly correlated to the increase in the mean knowledge score about the causes of AMR and antibiotic resistance (p<0.001), suggesting that the intervention positively impacted the participants' understanding of antibiotic resistance. These findings are similar to what was reported in India [22], Malaysia [19], and Egypt [23]. The Malaysian study did a follow-up post-intervention for two weeks and found that the knowledge was sustained [19]. This indicates that educational intervention could be used as a long-term strategy to improve knowledge of AMR. Research showed a significant improvement in knowledge scores post-educational intervention, highlighting the efficacy of educational interventions in enhancing understanding of AMR [19]. Post-intervention, the knowledge of leftover antibiotics, not completing a full course of antibiotics, and the misuse of antibiotics without consulting as causes of antibiotic resistance were improved compared to pre-intervention. Similar findings were reported in Malaysia and Jordan [19,24]. The incomplete dose is one of the challenges related to nonadherence to medications that were shown to lead to AMR. A Jordanian study found that participants with poor knowledge of antibiotic use had a significantly higher rate of nonadherence (p<001) and reported a significant association between nonadherence and education (p<0.05) [24].

Aligning with previous studies [19,25], we found statistically significant improvement in the knowledge of antibiotic use before and after the education intervention (p<0.001) and between the two study groups (p<0.001). These results indicate that the intervention positively influenced participants' understanding of the link between antibiotic misuse and antibiotic resistance. In India and China, research showed that most respondents (75% and 61%, respectively) thought that viral infections like the flu and the common cold could be treated with antibiotics [11], which is false since antibiotics are antibacterial only. Our study showed that some participants believed the same and highlighted the effectiveness of reducing this misbelief by showing a statistically significant decrease in that belief mean score after the education intervention (p<0.001). Therefore, education should be enhanced to teach people the types of medications used for bacterial and viral infections, especially respiratory infections.

We also found a statistically significant decrease in the belief that taking antibiotics for common cough and wheezing and (p=0.003) taking a low dose of antibiotics is better for infection prevention (p<0.001). These misbeliefs could lead to increased inappropriate antibiotic use and subsequent resistance due to self-medication. We found that almost half of the participants in both groups used to self-medicate. A Polish study found that 23% of respondents expected to be prescribed antibiotics for a sore throat, and 41% expected them for the flu [25]. Another study conducted in the Middle East showed that upper respiratory tract infections were the main reasons for self-medication, and penicillin was the most commonly self-medicated drug [26]. Thus, our findings showed that the intervention could effectively address misconceptions regarding antibiotic usage, specifically curing viral infections and using antibiotics for common cough and wheezing.

There was a statistically significant difference (decrease) between the mean score before and after the education intervention (p=0.008) and between the two study groups (p=0.002) regarding the worry of antibiotic resistance. Previous studies in Malaysia, Vietnam, Indonesia, and China also reported worry among participants, but studies showed that they perceived themselves as incapable of doing anything to prevent antibiotic resistance [11,19]. Our study showed a decrease in worry, which might be attributed to an improved understanding of participants' role in curbing AMR. This indicates that education can improve perceptions of AMR and increase awareness of its effects on individuals and society. Research suggested that curricula should include specific modules on antibiotic use and AMR to engage students to potential future professionals in combatting AMR [27].

This study may be limited by the fact that it was conducted in urban areas, making it not well representative of the situation in rural areas. In addition, the sample size could have been increased. Therefore, future studies extending to more settings in different areas (both urban and rural) are essential to mitigate these limitations.

## Conclusions

This study showed that interventional education is effective in raising awareness, understanding, and perceptions of AMR. It showed that the intervention positively impacted the participants' understanding of AMR, influenced their knowledge regarding its causes, and influenced their understanding of the link between drug misuse and AMR resistance. These findings also demonstrated that the intervention successfully reduced the misconception about antibiotics, emphasizing the importance of targeted educational efforts to promote appropriate antibiotic use. By raising knowledge, educational interventions enable people to make wise decisions about using antibiotics, encourage responsible prescribing techniques, and aid AMR stewardship initiatives. Implementing and maintaining educational interventions across various settings and groups in Saudi Arabia is crucial for effectively combating the AMR threat.
